# Extrusion Pretreatment of Lignocellulosic Biomass: A Review

**DOI:** 10.3390/ijms151018967

**Published:** 2014-10-20

**Authors:** Jun Zheng, Lars Rehmann

**Affiliations:** Department of Chemical and Biochemical Engineering, University of Western Ontario, 1151 Richmond Street, London, ON N6A 3K7, Canada; E-Mail: jzheng23@uwo.ca

**Keywords:** lignocellulosic biomass, extrusion pretreatment, bioethanol, extruder

## Abstract

Bioconversion of lignocellulosic biomass to bioethanol has shown environmental, economic and energetic advantages in comparison to bioethanol produced from sugar or starch. However, the pretreatment process for increasing the enzymatic accessibility and improving the digestibility of cellulose is hindered by many physical-chemical, structural and compositional factors, which make these materials difficult to be used as feedstocks for ethanol production. A wide range of pretreatment methods has been developed to alter or remove structural and compositional impediments to (enzymatic) hydrolysis over the last few decades; however, only a few of them can be used at commercial scale due to economic feasibility. This paper will give an overview of extrusion pretreatment for bioethanol production with a special focus on twin-screw extruders. An economic assessment of this pretreatment is also discussed to determine its feasibility for future industrial cellulosic ethanol plant designs.

## 1. Introduction

Environmental, long term, economic and national security concerns associated with the use of fossil fuels have strengthened the interest in alternative, nonpetroleum-based sources of energy, such as sunlight, rain, wind energy and so on, in the past two decades [1]. Biomass appears to be the only suitable and renewable primary energy source that can provide alternative liquid transportation fuels. Currently, ethanol is being produced from sugar-and starch-based materials such as sugarcane and corn, but its sustainability has been considerably debated [2]. Another interesting alternative raw material for the second generation production of ethanol is from lignocellulosic biomass, which offers large-scale availability with low cost, limited conflict with food crops and lower fossil fuel inputs [3]. The main composition of lignocellulosic biomass is cellulose, hemicelluloses and lignin. Cellulose is the most abundant of biopolymers on earth [4,5]. A worldwide annual production of 1 × 10^10^ metric tine (t) was estimated [6]. Lignocellulosic feedstocks could be grouped into five main categories: (1) agricultural residues; (2) dedicated energy crops; (3) municipal solid wastes; and (4) forestry residues; (5) food processing and other industrial wastes. Ethanol from various lignocellulosic biomasses is now considered the most promising mid-term fuel-additive to be blended with gasoline in dedicated engines [7].

Lignocellulosic biomass can be converted into fermentable sugars for fermentative ethanol production. However, this bioconversion is further complicated due to recalcitrance caused by the association of cellulose, hemicelluloses and lignin in the biomass [8]. Cellulose chains are embedded in a cross-linked matrix of hemicellulose wrapped by lignin on the outside, making the sugars inaccessible to the enzyme [9]; therefore, pretreatment is required to reduce the recalcitrance of lignocellulosic biomass by opening or partially breaking up the recalcitrant structure, while minimizing the chemical degradation of fermentable sugars for enhancing the enzymes’ accessibility to the cellulose during the enzymatic hydrolysis step. In addition, the cost and efficiency of enzymatic hydrolysis and fermentation will be affected by pretreatment since it is the first major unit operation in the process of bioconversion. An effective pretreatment should have the following criteria: reducing cost and minimizing energy requirements, preserving hemicellulose fractions (mainly pentose), avoiding the degradation of sugars and minimizing the formation of inhibitors for further fermentation steps, and recovery of lignin for valuable co-products productions [10].

A large number of pretreatment methods have been proposed generally on a wide variety of lignocellulosic biomasses for bioethanol production since different feedstocks have different physical-chemical characteristics. These pretreatment methods are usually divided into physical, chemical, physical-chemical and biological, such as steam explosion [11], dilute acid pretreatment [12], organosol pretreatment [2] and alkali pretreatment [13]. Some articles have generally reviewed biomass pretreatment [14,15,16,17,18,19]; however, only a detailed review of extrusion pretreatment processes is missing in the current literature. The objective of this paper is to give an overview of the extrusion pretreatment along with the advances achieved in recent years that show a promising method for bioethanol production. An economic assessment of this pretreatment is also discussed to determine the feasibility for future industrial cellulosic ethanol plant designs.

## 2. Pretreatment of Lignocellulosic Biomass

### 2.1. Overview of Extrusion Pretreatment

Extrusion is defined as an operation of creating objects of a fixed, cross-sectional profile by forcing them through a die of the desired cross-section. The material will experience an expansion when it exits the die. The extrusion process has been expanded as one of the physical continuous pretreatment methods towards bioethanol production due to its significant improvements of sugar recovery from different biomass feedstocks. Extrusion pretreatment has some advantages over other pretreatments: (1) low cost and provides better process monitoring and control of all variables [20]; (2) no sugar degradation products [21]; (3) good adaptability to different process modifications [22]; and (4) high continuous throughput [22]. It seems therefore that extrusion pretreatment is more feasible for the pretreatment of lignocellulosic biomass towards bioethanol production.

A screw extruder is based around screw elements, including (1) forward screw elements, which principally transport bulk material with different pitches and lengths with the least degree of mixing and shearing effect; (2) kneading screw elements, which primarily exert a significant mixing and shearing effect with different stagger angles in combination with a weak forward conveying effect; and (3) reverse screw elements, designed with a reverse flight to push the material backward, which carries out extensive mixing and shearing effects [23]. The arrangement of different pitches, lengths, stagger angles, positions and spacings define a screw configuration, which is a main factor influencing the extent of mixing, product transportation, extrudate properties, mechanical energy input and residence time distribution during extrusion processing [24,25,26,27]. With different screw configurations, the twin screw extruder can conduct diverse functions and processes in a single step, such as material transporting, heating, mixing, shearing, grinding, chemical reaction, drying and liquid-solid separation [28].

Different types of extruders, such as single-screw extruders and twin-screw extruders, have been widely examined for different lignocellulosic biomass, resulting in subsequently high enzymatic hydrolysis rates. The extrusion pretreatment process can be used as a physical pretreatment for the bioconversion of biomass to ethanol production; it also can be conducted in a large number of systems with or without the addition of chemical solutions. Karunanithy and Muthukumarappan [29,30] report that extruder parameters, such as compression ratio, screw speed, and barrel temperature, had significant effects on sugar recovery. Extruders will provide high shear, rapid heat transfer, and effective mixing in a short residence time. The physical and chemical structure of the material will be disturbed and changed during the passage through the extruder barrel, resulting in a large specific area to increase the accessibility of cellulose for enzymatic action [31]. An extrusion pretreatment can also be combined with other treatments to improve sugar recovery. Zheng *et al.* [32] evaluated an extrusion process with a dedicated filtration device after steam explosion treatment of corncobs for hemicelluloses separation.

Lignocellulosic biomass can be treated with chemical solutions such as acid and alkali during the extrusion process [33,34,35]; however, the acid would cause corrosion problems due to the construction material of the extruder, thus, acid resistant stainless steel alloys such as AL6XN, would be required for extruder screws and barrel fabrication [34,35]. Alkali pretreatment is a preferred method due to its role as a delignification agent with less degradation of carbohydrates. Among different alkali employed on lignocellulosic biomass, such as sodium, potassium, calcium and ammonium hydroxides, Morrison [36,37] reports that sodium hydroxide is the most known alkali in lignocellulosic biomass pretreatment and can cleave ester linkages and solubilise some hemicelluloses and lignin. Alkali pretreatment can be conducted by soaking the biomass in a sodium hydroxide solution at room temperature or by adding it to the extruder using a volumetric pump. Table 1 summarizes different kinds of extrusion pretreatments of lignocellulosic biomass on sugar yields.

**Table 1:** Effects of different kinds of extrusion pretreatment of lignocellulosic biomass on sugar yields. Sugar yields presented in the table were produced from different feedstocks and achieved under different operating conditions, the basis of the percentage values can also vary between each study. The results show the typical range and are for reference purposes, detailed comparisons should only be drawn between highly similar conditions and materials.

Extrusion Pretreatment	Lignocellulose		Extruder		Extrusion Conditions				Sugar Yield		Reference
Physical Pretreatment	Corn cobs		Single-screw extruder		Screw speed: 75 rpm, temperature: 125 °C				Glucose: 75%, xylose: 49%, combined sugar: 61%		[38]
	Switchgrass, big bluestem, prairie cord grass		Single-screw extruder		Screw speed: 200, 200, and 150 rpm, temperature of 75, 150, and 100 °C, respectively				Combined sugar: 28.2% for switchgrass, 66.2% for big bluestem, 49.2% for prairie cord grass		[39]
	Switchgrass, prairie cordgrass		Single-screw extruder		Screw speed: 50 rpm, temperature: 150 °C, moisture content: 15% for switchgrass; screw speed: 50 rpm, temperature: 50 °C, moisture content: 25% for prairie cordgrass				Combined sugar: 45.2% for switchgrass Glucose: 61.4%, xylose: 84.3%, combined sugar: 65.8% for prairie cordgrass.		[30]
	Wheat bran, soybean hull		Twin-screw extruder		Screw speed: 7 or 3.7 Hz, temperature: 150 or 110 °C				Glucose: 41%–60%		[40]
	Big bluestem		Single-screw extruder		Screw speed: 150 rpm, temperature: 180 °C, moisture content: 20% wb, particle size: 8 mm				Glucose: 71.3%, xylose: 78.5%, combined sugar: 56.9%		[41]
	Switchgrass		Single-screw extruder		Screw speed: 155 rpm, temperature: 176 °C, moisture content: 20% wb, particle size: 8 mm				Glucose: 41.4%, xylose: 62.2% combined sugar: 47.4%		[29]
	Corn stover		Twin-screw extruder		Screw speed: 80 rpm, moisture content: 27.5%, enzyme dosage: 0.028 g enzyme/g dry corn stover				Glucose: 48.79%, xylose: 24.98%, combined sugar: 40.07%		[42]
	Soybean hulls		Twin-screw extruder		Screw speed: 350 rpm, temperature: 80 °C, moisture content: 40%				Glucose: 95%		[43]
	Douglas-fir		Batch-type kneader		Screw speed: 90 rpm, temperature: 40 °C				Glucose: 54.2%		[44]
	Miscanthus		Twin-screw extruder		Screw speed: 100 rpm, temperature: 100 °C, feed rate: 15–30 kg dry matter/h of biomass				Glucose: 69% and xylose: 38%		[21]
	Soy hull		Single-screw extruder		Screw speed: 60 rpm, temperature: 130 °C, moisture content: 12.5% wb				Glucose: 62.5%, xylose: 68.6%, combined sugar: 62.4%		[45]
Acid Pretreatment	Municipal solid wastes		Extruder type reactor				Temperature: 230 °C, pressure: 30–32 atm, pH value: 0.50, reaction time: 8–15 s		Glucose: 60%		[33]
	Pine sawdust		Twin-screw extruder		Screw speed: 110 rpm, temperature: 60 °C, head pressure: 780 psi, H2SO4 concentration: 70 wt %.				Glucose: 44.4%		[34]
	Pine sawdust		Twin-screw extruder		1, temperature: 130 °C, H2SO4 concentration: 5 wt %, reaction time: 25 min 2, temperature: 130 °C, H2SO4 concentration: 30 wt %, reaction time: 3 min				Glucose: 50% for case 1 Glucose:41% for case 2		[35]
	Rice straw		Twin-screw extruder		Screw speed: 40 rpm, temperature: 120 °C, H2SO4 concentration: 3 wt %.				Xylose: 83.7%		[46]
Alkali Pretreatment	Wheat straw		Extrusion type mixer		Screw speed: 35 rpm, temperature: 98 °C, 0.157 g/g biomass NaOH with 0.003 g/g biomass AHQ or 0.127 g/g biomass NaOH with 0.05 g/g biomass Na2S				Glucose: 90%–92%, lignin removal: 64%–72%, pentosans: 36%–43%		[47]
	Corn stover		Twin-screw extruder		Screw speed: 80 rpm, temperature: 140 °C, NaOH loading ratio: 0.04 g/g biomass				Glucose: 86.8%, xylose: 50.5%		[48]
	Switchgrass		Single-screw extruder		Screw speed: 118 rpm, temperature: 180 °C, particle size: 6 mm, NaOH concentration: 0.02 g/g biomass				Glucose: 90.5%, xylose: 81.5%, and combined sugar: 88%		[49]
	Big bluestem		Single-screw extruder		Screw speed: 155 rpm, temperature: 90 °C, particle size: 4 mm, NaOH concentration: 0.2 g/g biomass				Glucose: 90.1%, xylose: 91.5%, combined sugar: 89.9%		[50]
	Corn stover		Twin-screw extruder		Die temperature: 55–90 °C, ammonia loading: 0–2.0 g/g biomass				Digestibility: increased up to 32%, lignin reduction: 12.5%		[51]
	Corn stover		Twin-screw extruder		Screw speed: 325 rpm, temperature: 99 °C NaOH loading: 0.06 g/g biomass				Glucose: 83%, xylan: 89%, lignin removal: 71%		[52]
	Populus tremuloides		Twin-screw extruder		Screw speed: 124 rpm, NaOH concentration: 0.02 g/g biomass				Hemicellulose extraction: 90%		[53]
	Corn stover		Twin-screw extruder		Screw speed: 14–28 rpm, temperature: 190–220 °C, NaOH concentration: 0.06 g/g biomass				N/A		[54]
Alkali Combined Pretreatment	Douglas fir, Eucalyptus		Twin-screw extruder		Screw speed: 45–120 rpm, temperature: room temperature, combined with hot-compressed water				Glucose: 5 times higher than HCW treatment alone		[55]
	Corn cobs		Twin-screw extruder		Screw speed: 100 rpm, temperature: 100 °C, combined with steam explosion				Glucose: 88.41%, Xylose removal: 80%		[32]
	Prairie cordgrass		Single-screw extruder		Screw speed: 65 rpm, temperature: 90 °C, particle size: 8 mm, moisture content: 20%, combined with organic solvent fractionation				Glucose: 92%, xylan removal: 95%, lignin removal: 87%		[56]
	Bagasse		Twin-screw extruder		Screw speed: 15 rpm, temperature: 140 °C, combined with ionic liquids				Glucose: 90%		[57]

### 2.2. General Background of Single and Twin Screw Extrusion

The screw extruder is a well known technology in the production, compounding, and processing of plastics; it also can be used in food processing industries, such as pet food, cereals and bread. The single screw extrusion process consists of an Archimedean screw in a fixed barrel. It can be classified as a smooth barrel, grooved and/or pin barrel screw extruder. Both are employed when melting and pressure build up are required. However, the mixing ability of single screw extruders is limited to distributive mixing and dispersive mixing (spatial rearrangement of solids or fluids changes in physical properties, such as particle size reduction) [58]. These could be achieved by some types of twin screw extruders using relatively high shear stress screw elements, *i.e.*, kneading disks [59].

Twin-screw extruders consist of two parallel screws with the same length placed in a stationary barrel section. Twin-Screw extruders can be classified according to their direction of screw rotation, *i.e.*, co-rotating or counter-rotating for which the screws rotate in either similar or opposite directions, respectively. Twin-screw extruders can be further subdivided into fully, partial or non-intermeshing based on the relative position of the screws [60]. In contrast to a single screw extruder, the flights scrape the inside of the barrel, and at the same time, maintain a certain clearance between the barrel and screw. The fully intermeshing co-rotating extruders possess the channel, tip, lobal pools, apex and intermesh mixing regions that give rise to very high normal shear. However, the single screw extruder lacks intermesh and apex regions [61]. Therefore, the co-rotating, fully-intermeshing twin screw extruder is a dominant application for biomass processing [59].

## 3. Studies of Single and Twin Screw Extrusion for Biomass Pretreatment

### 3.1. Physical Pretreatment

Screw design strongly influences work done on the material and amount of shear force generated during extrusion processes such as compression ratio, screw speed and barrel temperature. Karunanithy and Muthukumarappan [38] conducted pretreatments through a single screw extruder by varying different extruder temperatures (25, 50, 75, 100, and 125 °C) and screw speeds (25, 50, 75, 100, and 125 rpm) on the pretreatment of corn cobs while varying enzymes and their ratios. As a result, 75%, 49% and 61% of glucose, xylose, and combined sugar recovery were obtained, respectively, at 75 rpm and 125 °C using a 1:4 cellulase and β-glucosidase combination. These results were 2.0, 1.7, and 2.0 times higher than the control sample. Similarly, Karunanithy *et al.* [39] investigated the effects of screw speeds (100, 150, and 200 rpm), barrel temperatures (50, 75, 100, 150, and 200 °C) and cellulase with β-glucosidase (1:1 to 1:4) on sugar yield from selected warm season grasses, such as switchgrass, big bluestem, and prairie cord grass. The highest values of 28.2%, 66.2% and 49.2% of combined sugar yield were obtained for switchgrass, big bluestem, prairie cord grass at screw speeds of 200, 200, and 150 rpm and at barrel temperatures of 75, 150, and 100 °C, respectively, when the ratio of cellulase and β-glucosidase was 1:4. The properties of biomass material also had significant effects on sugar recoveries. Karunanithy and Muthukumarappan [30] evaluated the effect of compression ratio (2:1 and 3:1), screw speed (50, 100, and 150 rpm), and barrel temperature (50, 100, and 150 °C) on the sugar recovery from switchgrass and prairie cordgrass while varying moisture contents (15%, 25%, 35% and 45% wb). The highest sugar recovery from switchgrass after enzymatic hydrolysis was 45.2% at a screw speed of 50 rpm, a barrel temperature of 150 °C with a moisture content of 15%. The maximum glucose, xylose and combined sugar recovery of 61.4%, 84.3% and 65.8% were achieved for prairie cordgrass at a screw speed of 50 rpm, a barrel temperature of 50 °C with a moisture content of 25%. In addition, both treatment conditions resulted in low concentration of glycerol and acetic acid (0.02–0.18 g/L) for both biomasses. Lamsal *et al.* [40] investigated two physical pretreatment methods, grinding and thermo-mechanical extrusion, on wheat bran and soybean hull. A higher reduction in sugar yield from extrusion was obtained compared to grinding for wheat bran, but not for soybean hulls. The best combinations of screw speed and barrel temperature were 7 Hz/150 °C and 3.7 Hz/110 °C leading to higher sugar yields. Later the effects of extrusion temperature (100, 110, 120 and 130 °C), screw speed (50, 60, 70 and 80 rpm) with three moisture contents (10%, 12.5% and 15% wb) for soy hulls were studied on a single screw extruder, resulting in 62.5%, 68.6% and 62.4% recoveries for glucose, xylose and combined sugars respectively at a barrel temperature of 130 °C, a screw speed of 60 rpm with moisture content of 12.5% wb were 1.7, 1.4 and 1.8 times higher than untreated samples [45]. Karunanithy and Muthukumarappan [41] optimized the extrusion pretreatment parameters of barrel temperature (45–225 °C) and screw speed (20–200 rpm) and big bluestem parameters (moisture content (10%–50% wb) while varying particle size (2–10 mm) for enzymatic hydrolysis for maximum sugar recovery using a single screw extruder. They recovered 71.3%, 78.5% and 56.9% of glucose, xylose, and combined sugars, respectively at a barrel temperature of 180 °C, a screw speed of 150 rpm, moisture content of 20% wb with particle size of 8 mm. As a result, 68.5% of surface area of the optimum pretreated big bluestem was increased compared to that of the control sample. Karunanithy and Muthukumarappan [29] also evaluated the effects of parameters of switchgrass such as particle size (2, 4, 6, 8, and 10 mm) and moisture content (10%, 20%, 30%, 40% and 50% wb) over a range of screw speeds (20–200 rpm) and barrel temperatures (45–225 °C) on a single screw extruder. The results show that moisture content, screw speed and barrel temperature had significant effects on sugar recoveries. They recovered 41.4%, 62.2% and 47.4%, of glucose, xylose, and combined sugar recoveries, respectively, at a barrel temperature of 176 °C, a screw speed of 155 rpm, moisture content of 20% wb with particle size of 8 mm. Zhang *et al.* [42] investigated the effect of the extrusion pretreatment method on corn stover and the intrinsic factors contributing to the improvement of sugar recovery, such as moisture content (22.5%, 25%, 27.5% wb) and screw speed (40–140 rpm) using a twin-screw extruder on sugar recovery. The maximum glucose, xylose, and combined sugar recovery were 48.79%, 24.98% and 40.07%, respectively at 27.5% of moisture content with a screw speed of 80 rpm and an enzyme dosage of 0.028 g enzyme/g dry corn stover. These results were 2.2, 6.6 and 2.6 times higher than that of untreated corn stover, respectively. The cellulose network was exposed because of the lignin destruction and the specific surface area of pretreated materials was significantly amplified over the control samples. Yoo *et al.* [62] conducted a thermo-mechanical pretreatment process on soybean hulls and compared with two traditional pretreatment methods, dilute acid and alkali hydrolysis. By comparison, 95% cellulose was converted to glucose when the optimum processing conditions were set up at a screw speed of 350 rpm, a barrel temperature of 80 °C with 40% moisture content in the soybean hulls. The conversion from cellulose to glucose was increased by 69.5%, 128.4% and 132.2% for dilute acid, alkali and extrusion pretreatments, respectively. Lower fermentation inhibitors in the extrusion pretreated substrate such as furfural, 5-(hydroxymethyl-2-furaldehyde (HMF), were found than those reported from the acid hydrolyzed substrate.

Fibrillation of Douglas-fir was performed using water with mechanical kneading forces instead of chemicals for biomass pretreatment in a batch type kneader with twin screw elements. Douglas-fir was milled in a ball milling for 20 min and then kneaded at 40 °C at 90 rpm in a batch-type kneader by adding water for 30 min. The results showed the surface area of cellulose was increased and the glucose yield from the fibrillated products by enzymatic hydrolysis was 54.2%, much lower than the extrusion process with chemicals [44].

### 3.2. Acid Pretreatment

Extruders can be used as an acid hydrolysis reactor. Acid pretreatment is effective for converting cellulose and hemicelluloses into monomeric sugars. For example, an extruder type reactor was used for dilute acid hydrolysis for municipal solid wastes and the optimal glucose yields reached 60% at temperatures of 230 °C, pressures of 30–32 atm, pH values of 0.50 and reaction times of 8–15 s [33]. A twin screw extruder reactor was also used for concentrated-acid hydrolysis of pine sawdust to break down and convert cellulose into low molecular weight carbohydrates. Experiments were controlled at 110 rpm screw speed, 60 °C barrel temperature, 780 psi head pressure and sawdust to acid feed rate ratio of 1 to 0.8 (70 wt % H_2_SO_4_). Consequently, 38.2% of dry sawdust solids were converted to soluble liquids and 44.4% of cellulose was converted to soluble monomer sugars and oligosaccharides [34]. Later the same operating conditions were applied with different acid concentrations (5 to 30 wt %) and temperatures (110, 120 and 130 °C). An acid-resistant stainless alloy, AL6XN, was used for extruder screws and barrel fabrication. As a result, 50% of theoretical glucose was achieved at a temperature of 130 °C and 5 wt % in 25 min and 41% of the theoretical glucose was converted in three minutes at a temperature of 130 °C with more concentrated acid conditions of 30 wt %. [35]. The twin screw extrusion process also can be combined with the hot water extraction process at a bench scale to prepare monomeric xylose hydrolysate. The effects of screw speed (30–150 rpm), barrel temperature (80–160 °C) and dilute sulfuric acid (1–3 wt %.) of the twin screw extruder was evaluated on the structural properties of extruded rich straw, sugar concentration and conversion rate, after which 83.7% of the xylan was converted to monomeric xylose when the optimal conditions of the extruder process was at 120 °C and 40 rpm with 3% sulphuric acid and the optimal condition of the extraction was 130 °C for 20 min. Finally, an 80% yield of the total saccharification was achieved after enzymatic hydrolysis [46].

### 3.3. Alkali Pretreatment

Alkali pretreatment can be performed at a lower temperature and pressure compared to other chemical pretreatment methods. The process in the extruder also does not cause as much sugar degradation [17]. Carr and Doane [47] employed an extrusion type mixer as a pretreatment method for wheat straw in order to disrupt the lignin-hemicellulose and cellulose complex. The milled wheat straw was treated with various chemical solutions, such as anthraquinone, anthrahydroquinone, hexamethylenediamine, hexamethylenetetramine, sodium hydroxide, ferrous ammonium sulfate and sodium sulfide. These chemicals were metered into the barrel by a high-pressure diaphragm. They acted as delignification agents without degrading the carbohydrates, so that 64%–72% of lignin and 36%–43% of the pentosans were achieved when these chemicals were used alone or in selected combinations. The conversion rate from cellulose to glucose reached 90%–92% at catalyst loadings of 0.157 g/g (NaOH), 0.003 g/g (antrahydroquinone (AHQ)), 0.127 g/g (NaOH), or 0.05 g/g (Na_2_S). Similarly, Zhang *et al.* [48] also evaluated the effect of twin screw extrusion to promote sugar yields from corn stover with different concentrations of alkali loadings (0.004, 0.012, 0.013 and 0.04 g/g biomass). Corn stover was mixed with alkaline solutions in a mechanical mixer and then fed into the extruder. The optimum glucose and xylose sugar yields were 86.8% and 50.5%, respectively, at an alkali loading ratio of 0.04 g/g biomass and screw speed of 80 rpm. These results were 3.9 and 13.3 times higher than the raw material. In addition, the alkali combined extrusion process produced more pores. Karunanithy and Muthukumarappan [49] also carried out pretreatments through a single screw extruder by varying different extruder parameters for maximum sugar recovery from switchgrass. The maximum glucose, xylose, and combined sugar recovery were 90.5%, 81.5% and 88%, respectively, at the optimized conditions of 180 °C barrel temperature, 118 rpm screw speed, 0.02 g/g (alkali/biomass), and particle size of 6 mm. Similarly, Karunanithy and Muthukumarappan [50] evaluated the combined effect of alkali soaking and extrusion on big bluestem using a single screw extruder. Big bluestem was soaked in different alkali concentrations at room temperature for 30 min and then extruded while varying barrel temperatures (45–225 °C) and screw speeds (20–200 rpm) and particle size of big bluestem (2–10 mm). Consequently, 90.1%, 91.5% and 89.9% of glucose, xylose and combined sugar were recovered, respectively, at a barrel temperature of 90 °C, a screw speed of 155 rpm, 0.2 g/g (alkali/biomass) with particle size of 4 mm. Dale *et al.* [51] conducted the extrusion pretreatment of corn stover with an ammonia solution injection on a twin screw extruder. The technique takes advantage of explosive depressurization resulting in a rapid expansion of liquid ammonia gas that disrupted biomass fibres. The digestibility was increased up to 32% over that of completely untreated material and 23% over extruded material without an ammonia solution. An average of 12.5% in lignin reduction was achieved from extrusion treated material. Liu *et al.* [52] performed the alkaline twin screw extrusion pretreatment of corn stover for fermentable sugar production with a biomass/liquid ratio of 1/2 (wt) at a temperature of 99 °C. They used 0.06 g/g biomass of NaOH and converted 83% of glucan and 89% of xylan, respectively; in addition, 71% lignin removal was achieved. N’Diaye *et al.* [53] employed a modified Clextral twin screw extruder to extract hemicelluloses through a filter from the hardwood Populus tremuloides; they added 0.02 g/g of a sodium hydroxide solution using a volumetric pump and the screw speed was fixed at 124 rpm for all experiments, subsequently, 90% of the initial hemicelluloses were extracted. Later the same application was performed using a continuous pilot-scale biomass fractionation extrusion process on corn stover. The Countercurrent and cocurrent process fractionates corn stover into three streams. Countercurrent prehydrolysis of hemicelluloses at 210 °C mainly extracted hemicellulosic sugars and cocurrent flow catalyzed by NaOH (0.06 g/g biomass) at 220 °C was used to remove lignin. The overall process was employed to produce a pure cellulose stream and low-molecular weight lignin [54].

### 3.4. Combined Pretreatment

Biomass extrusion can be utilized as a stand-alone pretreatment method, or in combination or sequence with other pretreatment techniques. Lee *et al.* [55] evaluated extrusion processes after hot-compressed water (HCW) treatment of Douglas fir and Eucalyptus. HCW effectively removed hemicelluloses and lignin while the extrusion process improved the micro/nano fibrillation. Douglas fir and Eucalyptus were pretreated under temperature ranging from 140 to 180 °C at a pressure of 1 MPa. The reaction time was 30 min and the water to starting material weight was 5:1. The water insoluble residue was then subjected to a twin-screw extruder at room temperature with a screw speed of 45–120 rpm. The produced glucose levels were five times higher than those obtained by HCW treatment alone. In a different study, Miscanthus was pretreated with sequential mechanical (extrusion) and chemical pretreatment (sodium hydroxide). The extruder conditions were 100 rpm screw speed, 15–30 kg dry matter/h of biomass throughput and 100 °C barrel temperature. The extrusion treated Miscanthus was then pretreated with 12% NaOH (wt dry matter) at different solid/liquid ratios at 70 °C for 4 h. The combination pretreatment resulted in 77% delignification. Following this, 69% and 38% of the initial cellulose and hemicelluloses were converted into glucose, xylose and arabinose, respectively [21]. Zheng *et al.* [32] evaluated a modified twin-screw extruder incorporated with a dedicated filtration device after steam explosion. The ground corncobs were pretreated by steam explosion, and hemicelluloses were hydrolyzed largely to xylose. Subsequent solid liquid separation in a twin-screw extruder resulted in the removal of 80% xylose along with other inhibitors such as soluble lignin (100 rpm screw speed, 100 °C barrel temperature, mass flow rate of 4 kg/h). Enzymatic saccharification of the remaining solids resulted in 88% glucose conversion was. Similarly, Brudecki *et al.* [56] investigated the effects of sequential extrusion and clean fractionation processing. Ground Prairie Cordgrass was extruded in a single screw extruder under the optimized conditions (90 °C barrel temperature, 65 rpm screw speed, 20% moisture content, 8 mm particle size). A clean fractionation process was implemented after the extrusion process to fractionate the extruded material into cellulose, hemicelluloses and lignin fractions. Different proportions of organic solvent mixtures including methyl isobutyl ketone (MIBK), ethanol and water with sulfuric acid were used. Consequently, 92% glucose was yielded, and 87% lignin and 95% xylan were removed under the optimal conditions of 39 min, 129 °C, 0.69% catalyst and 28% MIBK. Ionic liquids are considered as promising solvents for lignocellulosic biomass pretreatment due to their unique solubilization properties. A combination of extrusion in the presence of ionic liquids was performed [57]. The extrusion process was carried out using a twin-screw extruder at 140 °C with a screw speed of 15 rpm in the presence of 1-ethyl-3-methylimidazolium acetate ([Emim][Ac]. Different bagasse loadings were examined and the results showed that more than 90% glucose was yielded after 24 h of enzymatic hydrolysis from the pretreated baggases at a loading of 25 wt % for 8 min at 140 °C [57].

## 4. Economic Analysis of Extrusion Pretreatment Methods

Many pretreatment technologies of lignocellulosic biomass have been studied to improve ethanol yield [63]. Thermo-mechanical extrusion has been used for lignocellulosic biomass pretreatment, resulting in a higher efficiency of enzymatic hydrolysis. An economic, feasible assessment of the extrusion pretreatment methods should be evaluated in order to design an industrial bio-ethanol production plant. However, a comparison of the economic feasibility of each pretreatment is very difficult due to different underlying assumptions [64]. Kazi *et al.* [65] carried out a quantitative data analysis of different pretreatment technologies for the production of ethanol from lignocellulosic materials, including dilute acid, 2-stage dilute-acid, hot water, and ammonia fibre explosion (AFEX). This analysis was based on a short-term commercial viability of biochemical ethanol production and each pretreatment process was embedded in a full facility model. Publicly available experimental data was used for the total capital investment and product value estimation. Biomass corn stover was used for all pretreatment technologies in the analysis. An ASPEN Plus simulation model of a full bio-ethanol production facility was used for each pretreatment model. In the dilute-acid pretreatment, concentrated sulfuric acid was diluted to 1.1% and the pretreatment reactor operated at 12.1 atm pressure and 190 °C. In the 2-stage dilute-acid pretreatment, the first stage solubilizes most of the hemicelluloses and dilute acid hydrolyzes a fraction of cellulose and remaining hemicelluloses in the second stag. In the hot water pretreatment, the corn stover was chopped and washed from the pretreatment section and mixed with hot water in a plug flow pretreatment reactor when the pressure and temperature were maintained at 12.5 bars and 190 °C, respectively, for a residence time of 5 min. In the AFEX pretreatment, the corn stover was treated with liquid ammonia, where the pressure was maintained at 17.2 bars and the temperature was held at 60 °C for 5 min; the pressure was released rapidly to make the fibres explode. The analysis in terms of pretreatment cost on total capital, installed equipment investment and the ethanol annual capacity, yield and product value are given in Table 2. The dilute-acid pretreatment had the highest ethanol yield (289 L/t while the ethanol yield for other pretreatments varied between 177 and 250 L/t). In addition, the dilute-acid pretreatment has the lowest product value of $1.36/LGE (liter of gasoline equivalent) among all pretreatments.

**Table 2:** Techno-economic analysis for each pretreatment technology. Adapted from [65] with permission form Elsevier, copyright 2010.

Pretreatment Method	Total Capital Investment ($ Million)	Total Installed Equipment Cost ($ Million)	Ethanol Production Million/a	Ethanol Yield (L/t)	Product Value ($/LGE)
Dilute acid	376	164	202	289	1.36
2-Stage dilute-acid	391	173	124	177	1.75
AFEX	386	167	175	250	1.47
Hot water	327	156	148	211	1.77

LGE: Liter of gasoline equivalent; PV: Ethanol production cost, including a 10% return on investment.

Yoo *et al.* [43] analyzed the technical and economic competitiveness of thermo-mechanical extrusion pretreatment with dilute acid hydrolysis for cellulosic ethanol production. The Monte Carlo simulator model was employed to estimate the sugar yield and production cost over a year of production. Biomass soybean hull was used as raw material in the analysis. In the dilute acid hydrolysis, the biomass was run through in three reactor trains consisting of a presteamer, blow tank, and reactor, and the solids coming from the reactor were separated from the slurry using a pressure filter for enzymatic saccharification [66]. In the extrusion process, the biomass was hydrolyzed and softened by steam and water in a preconditioner, and wet extruded pellets were mixed with additional water in a tank for further enzymatic saccharification. The production cost of each pretreatment was performed at the plant scale under some well-defined assumptions. The results of total fixed capital, pretreatment direct fixed capital and the ethanol production of each pretreatment are given in Table 3. As a result, 53.7 million gal/year of ethanol was produced from the extrusion process, which was around 23.4% more ethanol than that produced from the acid hydrolysis process at 43.5 million gal/year; this is due to the higher glucose conversion efficiency from the extrusion pretreatment process. Yoo *et al.* [43] report that 94.8% and 69.2% cellulose to glucose conversion was achieved utilizing extrusion and dilute acid hydrolysis pretreatments, respectively. However, if the feedstock was replaced by corn stover, the amount of hemicelluloses would be two times higher than the amount of soybean hulls, and thus more pentose could be fermented to cellulosic ethanol. The total fixed capital investment for each pretreatment was 174 and 191 million, respectively, and the total pretreatment equipment capital costs for extrusion and dilute acid hydrolysis were estimated at about 25 and 27 million, respectively, due to the fact that the extrusion process is simpler and required less equipment. From the results, its proven that extrusion pretreatment is a promising pretreatment technology for cellulosic ethanol production compared to dilute acid hydrolysis due to lower capital, pretreatment cost and higher sugar conversion efficiency [43].

**Table 3:** Capital cost for each pretreatment technology [43].

Pretreatment	Pretreatment Direct Fixed Capital, Million $	Total Fixed Capital, Million $	Ethanol Production Million gal/a	Total Fixed Capital $/gal Annual Capacity
Dilute acid	27.0	191	43.5	4.39
Extrusion	25.0	174	53.7	3.24

The underlying assumptions of each economical model have to be carefully evaluated when comparing different pretreatment techniques, as can be illustrated by the different assumptions for the capital costs of a diluted acid process of 376 MM by Kazi *et al.*, 2010 [65] and 191 MM by Yoo *et al.*, 2011 [62] made in different years for otherwise similar projects. Ultimately, no commercial cellulosic ethanol plant has been built to date using extrusion pretreatment. The Beta Renewables Company started the world’s first commercial cellulosic ethanol plant in Italy with a production capacity of 75 million liters in 2013. The plant is based on the steam explosion and hot water pretreatments from local wheat straw, rice straw and Arundo donax. Few companies have started to test cellulosic ethanol plants on a demonstration scale using some pretreatment methods, such as steam explosion, liquid hot water and dilute acid hydrolysis (Table 4) [67]. The PureVision Technology employs a modified continuous countercurrent extruder reactor (CCER) to rapidly fractionate biomass, such as corn stover and wheat straw, into xylose, lignin, and a digestible solid cellulose fraction that can be converted to glucose in minutes. It yields 80% of total mixed sugars. The company is now proceeding from its experimental concepts to its half-a-tonne-a-day continuous extruder reactor. The first commercial scale 250 tons per day CCER will be built in 2014 [68].

**Table 4:** Worldwide production of bioethanol. Adapted with permission from Dina *et al.* [67].

Company	Location	Products	Status	Raw Material	Pretreatment
Abengoa	Spain	4000 t/a EtOH	Demo facility, operational since 2008	Wheat straw, barley, corn stover	Acid catalyzed steam explosion
Clariant	Germany	1000 t/a EtOH	Demo facility, operational since 2012	Agriculture residues, wheat straw	Thermo-mechanical
Inbicon	Denmark	4300 t/a EtOH	Demo facility, operational since 2009	wheat straw	Hydrothermal (Liquid hot water)
Blue Sugar Corporation	USA	4500 t/a EtOH	Demo facility, operational since 2008	Sugarcane bagasse and other biomass	Thermo-mechanical
BP Biofuels	USA	4200 t/a EtOH	Demo facility, operational since 2009	Dedicated energy crops	Biochemical (steam explosion and mildly acidic conditions)
Iogen	Canada	1600 t/a EtOH	Demo facility, operational since 2004	Straw (wheat, barley, oat), corn stover	Modified steam explosion

## 5. Conclusions

The main purpose of pretreatment is to remove hemicelluloses and lignin, to increase the accessible surface area for enzymes and to descrystallize cellulose. The advantages of extrusion pretreatment technologies have been listed and discussed above. However, due to the varying types of biomass, efficient and economical methods need to be developed in a feedstock-specific manner; here, a few reports of quantitative economical analyses of data of different pretreatment technologies were discussed. In this review, the feasibility and economical analysis of extrusion pretreatment offers an initial glimpse into future investigations in pretreatment technology. None of the cellulosic ethanol from extrusion pretreatment technology has been commercialized to date, and uncertainties and limitations are unavoidable in the economic analysis and comparison of conversion technologies. Therefore, identifying the economic impact of different pretreatments related to productivity, capital cost, and operating cost, as well as defined assumptions, is important when conducting an economic analysis of bioethanol to aid reliable and creditable cost predictions. Further, improvements in pretreatment, enzymatic hydrolysis, and fermentation should be studied in order to reduce production costs.
